# High temperature induces transcriptomic changes in *Crassostrea gigas* that hinder progress of ostreid herpesvirus (OsHV-1) and promote survival

**DOI:** 10.1242/jeb.226233

**Published:** 2020-10-15

**Authors:** Lizenn Delisle, Marianna Pauletto, Jeremie Vidal-Dupiol, Bruno Petton, Luca Bargelloni, Caroline Montagnani, Fabrice Pernet, Charlotte Corporeau, Elodie Fleury

**Affiliations:** 1 Ifremer, Université de Brest, CNRS, IRD, LEMAR, F-29280 Plouzané, France; 2 Cawthron Institute, 98 Halifax Street East, Private Bag 2, Nelson 7042, New Zealand; 3 Department of Comparative Biomedicine and Food Science, University of Padova, Viale dell'Università 16, 35020 Legnaro, Padova, Italy; 4 IHPE, Université de Montpellier, CNRS, Ifremer, Université de Perpignan, Via Domitia, F-34095 Montpellier, France

**Keywords:** Anti-viral molecular pathway, Host–pathogen interaction, Marine disease, OsHV-1, Resistance, Temperature

## Abstract

Of all environmental factors, seawater temperature plays a decisive role in triggering marine diseases. Like fever in vertebrates, high seawater temperature could modulate the host response to pathogens in ectothermic animals. In France, massive mortality of Pacific oysters, *Crassostrea gigas*, caused by the ostreid herpesvirus 1 (OsHV-1) is markedly reduced when temperatures exceed 24°C in the field. In the present study we assess how high temperature influences the host response to the pathogen by comparing transcriptomes (RNA sequencing) during the course of experimental infection at 21°C (reference) and 29°C. We show that high temperature induced host physiological processes that are unfavorable to the viral infection. Temperature influenced the expression of transcripts related to the immune process and increased the transcription of genes related to the apoptotic process, synaptic signaling and protein processes at 29°C. Concomitantly, the expression of genes associated with catabolism, metabolite transport, macromolecule synthesis and cell growth remained low from the first stage of infection at 29°C. Moreover, viral entry into the host might have been limited at 29°C by changes in extracellular matrix composition and protein abundance. Overall, these results provide new insights into how environmental factors modulate host–pathogen interactions.

## Abstract (precis)

**Summary:** New insights into the mechanisms underlying the high survival of oysters infected with OsHV-1 at 29°C, inducing host physiological processes that are unfavorable to the viral infection.

## INTRODUCTION

Mortality outbreaks in the Pacific oyster, *Crassostrea gigas*, associated with infection by viral and bacterial pathogens have increased during the last 10 years worldwide (Barbosa Solomieu et al., 2015; EFSA, 2010; Pernet et al., 2016). The most striking example is the massive mortality of individuals less than 1 year old, which can decimate up to 100% of farmed oysters during the warm season. These mortalities coincided with the recurrent detection of ostreid herpesvirus 1 (OsHV-1) variants (Jenkins et al., 2013; Lynch et al., 2012; Mortensen et al., 2016; Segarra et al., 2010). The virus induces an immune-compromised state in oysters, evolving towards subsequent bacteremia by opportunistic bacterial pathogens leading to oyster death (De Lorgeril et al., 2018). Concomitantly, like other herpesviruses, OsHV-1 uses the host cell machinery to replicate (Jouaux et al., 2013; Renault and Novoa, 2004; Segarra et al., 2014a) and alters its metabolism (Corporeau et al., 2014; Pernet et al., 2019; Young et al., 2017).

Seawater temperature is a major trigger of marine disease, influencing the host and the pathogen (Burge et al., 2014; Harvell et al., 2002; Rahman et al., 2006). Temperature modulates host physiology by altering the velocity of chemical and enzymatic reactions, rates of diffusion, membrane fluidity and protein structure (Hochachka and Somero, 2002; Pernet et al., 2007). Previous studies showed that thermal stress induces a beneficial expression of immune-related genes in oysters (Green et al., 2014; Zhang et al., 2015). For instance, 9-month-old oysters respond more vigorously to a virus-associated molecular pattern at 22°C compared with 12°C, increasing the levels of TLR (Toll-like receptor), MyD88 (myeloid differentiation response 88), Rel (C-REL), MDA5 (melanoma differentiation-associated protein 5), IRF (interferon regulatory factor) and viperin in their hemocytes (Green et al., 2014).

Regarding OsHV-1, the optimal seawater temperature in Europe for disease transmission and subsequent mortalities is between 16 and 24°C (Pernet et al., 2012; Renault et al., 2014). In a previous study, we found that survival of oysters challenged with OsHV-1 at 29°C was markedly higher (85.7%) than at 21°C (52.4%), whereas virus infectivity and virulence were unaltered (Delisle et al., 2018). We therefore hypothesized that the key factor affecting survival rate was the host response to the pathogen rather than the virus itself. To test this hypothesis, we characterized the physiological condition of oysters at 21 and 29°C, assessed the amount of OsHV-1 in oysters and then compared their transcriptomes during the course of infection. In contrast to previous studies which describe the mechanisms of infection under permissive conditions by comparing healthy versus infected (Jouaux et al., 2013; Rosani et al., 2015) or resistant versus susceptible populations (De Lorgeril et al., 2018; Segarra et al., 2014a), we investigate the physiological mechanisms that modulate the severity of the disease by infecting susceptible oysters at two temperatures that are more or less permissive.

## MATERIALS AND METHODS

### Experimental design

Details about the rearing procedures, experimental design and samples are presented in a previous paper (Delisle et al., 2018). Briefly, specific pathogen-free oysters, *Crassostrea gigas* (Thunberg 1793), were produced in a hatchery at the Ifremer facilities in Argenton according to Petton et al. (2015). Prior to starting the experiments, young oysters were maintained in a 500-liter open flow tank under controlled conditions (21°C, 35.2‰ salinity, O_2_>85%), and fed *ad libitum*. At the onset of the experiment, oysters were 8 months old with a mean mass of 1.48±0.2 g. On 9 May 2016, oysters were divided in two groups: oysters for injection with a suspension of OsHV-1 (i.e. pathogen donors), or oysters for co-habitation with pathogen donors (i.e. pathogen recipients). Pathogen donors were left at 21°C (control temperature) in 500-liter tanks while recipient oysters were transferred to 45-liter tanks either left at 21°C or gradually increased to 29°C at 2°C day^−1^ (*N*=3 replicate tanks per temperature). On 19 May 2016, oysters for injection were myorelaxed in hexahydrate MgCl_2_ (30 g l^−1^), as described by Suquet et al. (2009). Oysters were injected in the adductor muscle with 100 μl of viral suspension containing 6.9×10^6^ copies of OsHV-1 μVar (Schikorski et al., 2011) and incubated at 21°C for 5 h. Injected oysters (now pathogen donors) were then transferred into the 45-liter tanks to co-habit with the recipients acclimated at 21 or 29°C. Survival of recipients was assessed every day for 14 days (Delisle et al., 2018), and 15 recipients per tank were sampled at 0, 12, 24, 48 and 96 h post-cohabitation (hpc). Whole oyster tissues were removed from the shell, frozen in liquid nitrogen and individually ground in liquid nitrogen with a MM400 homogenizer (Retsch, Eragny, France). One individual oyster per tank was used for both transcriptomic analyses and OsHV-1 DNA quantification (*N*=3 replicate tanks per temperature) and one pool of nine oysters was used for biochemical analysis.

### OsHV-1 DNA quantification

Level of OsHV-1 DNA was quantified in one whole oyster per tank (*N*=3 replicate tanks per temperature), sampled at 0, 12, 24 and 48 hpc. These analyses were conducted by Labocea (Brest, France) using oyster powder homogenized in sterile artificial seawater (Pepin et al., 2008). Total DNA was then extracted with a QIAamp tissue mini kit (Qiagen, Hilden, Germany) according to the manufacturer's protocol. The extract was stored at −20°C before detection and quantification according to a real-time PCR protocol based on SYBR Green chemistry (Pepin et al., 2008) with specific primers previously validated (Webb et al., 2007). The results were expressed as the log of OsHV-1 DNA copies per milligram of wet tissue.

### RNA extraction and RNA sequencing

Total RNA was isolated using the Direct-Zol RNA MiniPrep kit (Proteigene, Saint Marcel, France) according to the manufacturer's protocol. Total RNA was treated with Turbo DNAse (Ambion, Austin, TX, USA) to remove genomic DNA. The RNA quality and quantity were determined using NanoDrop 2000 (Thermo Fisher Scientific, Waltham, MA, USA) and Bioanalyzer 2100 (Agilent Technologies, Santa Clara, CA, USA). All samples complied with purity (OD260/OD280 and OD260/OD230>1.8) and quality criteria (RNA integrity number >8) required for cDNA library preparation. A total of 30 cDNA libraries were synthesized and sequenced (two temperatures, five sampling times, in triplicate).

RNA-seq library construction and sequencing were performed by Eurofins Genomics (Nantes, France). Directional cDNA libraries were constructed using a TruSeq Stranded mRNA kit (Illumina, San Diego, CA, USA) and sequenced on a Hiseq4000 in paired end reads of 2×125 bp.

#### Reads processing

Initial quality control was carried out with FastQC software (http://www.bioinformatics.babraham.ac.uk/projects/fastqc/), version 0.11.5 (Andrews, 2010). In order to filter out any remaining post-sequencing ribosomal RNA, the local sequence alignment tool SortMeRna 2.0 (Kopylova et al., 2012) was applied against different public databases (Rfam 5.8S; Rfam 5S; Silva 16S archaeal, bacterial; Silva 18S eukaryote; Silva 23S archaeal, bacterial; Silva 28S eukaryote) and a custom database including *C. gigas* ribosomal RNAs. Raw reads were then trimmed for low quality bases using CLC Genomics Workbench version 11.0 as follows: (1) Illumina adapters were removed; (2) only reads with less than two ambiguous nucleotides were allowed; (3) reads with >5% nucleotide with PHRED scores <20 were filtered out; (4) broken pairs were discarded.

#### Reads mapping

Reads were mapped against the Ensembl *C. gigas* reference genome version 9.38 by means of the STAR aligner and following the two-pass mapping mode (Dobin and Gingeras, 2015). The maximum number of mismatches allowed was set to 20 and only uniquely mapped reads were counted. Read counts for each sample, at the gene level, were extracted by setting the ‘GeneCounts’ quantification while running STAR. A summary of the metrics of the reads obtained is available from Dryad (Table S1; https://doi.org/10.5061/dryad.m37pvmcz0).

#### Differential expression analysis

Extracted read counts were used for the analysis of differential gene expression, which was conducted using the Bioconductor package edgeR version 3.10.0 (Robinson et al., 2010) in the R environment (version 3.2.2). Samples were grouped according to condition, temperature and time. The edgeR ‘calcNormFactors’ normalization function was used to find a set of scaling factors for the library sizes that minimized the log-fold changes between samples. The scale factors were computed using a trimmed mean of M-values (TMM) between samples (Robinson et al., 2010). After estimating dispersions, the ‘glmLRT’ test provided in edgeR was used to assess differential expressed genes (DEGs) between experimental conditions, with a threshold for a significant false discovery rate (FDR) set to <0.05.

#### GO term enrichment analysis

A functional interpretation of the lists of significant genes was obtained through enrichment analysis using the Database for Annotation, Visualization, and Integrated Discovery (DAVID) software (https://david.ncifcrf.gov/home.jsp). ‘Biological process’ (BP) annotation category (BP_FAT) was used by setting the gene count equal to 3 and the ease value equal to 0.05. As the DAVID database contains functional annotation data for a limited number of species, it was necessary to link the *C. gigas* genes with sequence identifiers that could be recognized in DAVID. This process was accomplished using Uniprot/Swiss-prot accession IDs corresponding to each contig. These identifiers were used to define different ‘gene lists’ of DA transcripts (up- and down-regulated separately) and a ‘background’ (all the expressed genes) in the bioinformatic tool DAVID. Heatmaps were constructed using Multiple Experiment Viewer software (Saeed et al., 2003; http://mev.tm4.org/#/datasets/upload).

### Biochemical analysis

#### Lipid class determination

Lipid class determination was obtained using 300 mg of oyster powder homogenized in 6 ml chloroform–methanol (2:1, v/v) according to Folch et al. (1957). Neutral and polar lipid class determinations were performed using a CAMAG automatic sampler (CAMAG, Muttenz, Switzerland) as described by Da Costa et al. (2016). These methods allowed the different lipid classes to separate into sterols (ST), alcohols (AL), alkenones 1 and 2 (ALK1 and -2), free fatty acids (FFA), triacylglycerol (TAG), glyceryl ethers (GE) and sterol esters (StE) for neutral lipids and lysophosphatidyl-choline (LPC), sphingomyelin (Sm), phosphatidylcholine (PC), phosphatidylserine (PS), phosphatidylinositol (PI), phosphatidylethanolamine (PE) and cardiolipin (Ca) for phospholipids.

#### Fatty acid composition

Fatty acid composition was analysed in neutral and polar lipids of oysters at 21 or 29°C at 0, 12, 24 and 48 hpc. Neutral and polar lipids were separated using a silica gel micro-column as described by Marty et al. (1992). Each lipid fraction was trans-esterified (Metcalfe and Schmitz, 1961) and analysed in a gas chromatograph with an on-column injector, DB-Wax capillary column and a flame ionization detector. Fatty acids were then identified by comparison of retention times with standards.

#### Carbohydrates

Samples of 50 mg of oyster powder were homogenized in 2 ml of Nanopure water using a Polytron PT 2500 E (Kinemetica, Luzern, Switzerland) and diluted 10 times. Carbohydrate concentrations were determined by the colorimetric method according to DuBois et al. (1956). Samples (250 µl) were mixed with phenol (0.5 ml, 5% m/v), and incubated for 20 min. Sulfuric acid (2.5 ml, 96%) was added to the samples. Absorbance was read at 490 and 600 nm with a UV 941 spectrophotometer (Kontron Instruments, San Diego, CA, USA). Carbohydrate concentrations were determined with the following formula: *A*=*A*_490_−1.5(*A*_600_−0.003), where *A* is absorbance (at 490 and 600 nm as indicated) and using a standard calibration curve. Concentration was expressed as milligrams of carbohydrates per gram of dry mass.

#### Protein and citrate synthase activity

Total protein extraction was performed as described by Guévélou et al. (2013) using 600 mg of oyster powder. Total protein concentration of each lysate was determined using the DC protein assay (Bio-Rad, Hercules, CA, USA). The resulting lysates were divided in aliquots and stored at −80°C until enzymatic assay.

All the assays were performed in triplicate at 21°C. Enzyme activity was measured using Nunc 96-well microplates (Thermo Fisher Scientific), a Synergy HT microplate reader and the software Gen5, both from Biotek (Winooski, VT, USA). Enzymatic activity was measured and related to the total protein concentration of each sample.

Enzymatic activity of citrate synthase (CS; EC 2.3.3.1) was measured using 20 μl of total protein lysates as described by Epelboin et al. (2015) and Fuhrmann et al. (2018).

### Statistical analyses

Mixed-design ANOVAs were performed to assess differences in (i) OsHV-1 DNA load in oysters depending on temperature (two levels, main plot) and time (four levels, subplot) and (ii) the proximate composition of oysters, activity of CS and fatty acid composition of polar lipids depending on temperature (two levels, main plot) at 48 hpc. The replication unit was the tank in which the temperature treatments were applied. All mutual interactions among factors were tested, and Tukey's honestly significant difference test was used as a *post-hoc* test. The normality of residuals and homogeneity of variances were graphically checked, and the data was log (*x*+1) transformed where necessary. Statistical analyses were performed in R, version 3.4.3 (R, Vienna, Austria; https://www.R-project.org/).

## RESULTS

### Survival

**Fig. 1.**
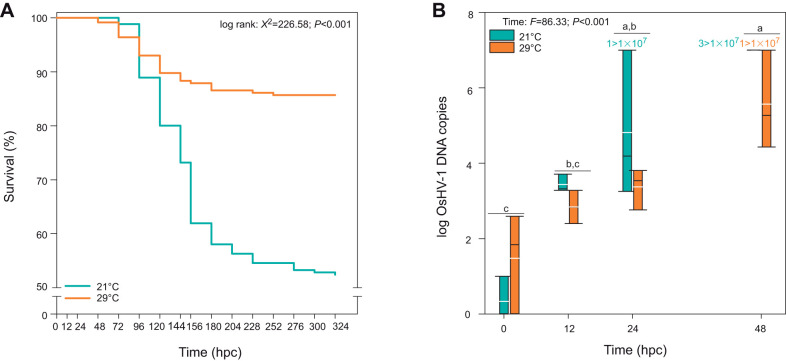
**Survival of OsHV-1 infected *Crassostrea gigas* oysters at 21 and 29°C and quantification of OsHV-1 DNA in oysters as a function of time at both temperatures.** (A) Survival of *Crassostrea gigas* oysters infected with OsHV-1 at 21 and 29°C. Survival time was measured in hours post-cohabitation (hpc) with pathogen donors. (B) Quantification of OsHV-1 DNA (copies mg^−1^) in oysters as a function of time at 21 and 29°C. Data were log(*x*+1) transformed, and lowercase letters indicate significant differences. The white and black lines within the bars represent the mean and median values, respectively. Numbers ‘1’ for 24 hpc and ‘3’ for 48 hpc indicate the number of oysters per condition that displayed OsHV-1 DNA value higher than 1.10^7^ copies per milligram.

The recipient oysters showed significant mortalities 72 h post-cohabitation (hpc) with donors at both tested temperatures. At this time, survival rates were 98.3±0.98% at 21°C and 96.4±1.8% at 29°C. At the end of cohabitation trial (14 days), the survival of oysters acclimated at 29°C was higher (85.7±2.0%) than at 21°C (52.4±3.1%; Fig. 1A).

### Virus load through infection course

At the onset of the experiment (0 hpc), low levels of OsHV-1 DNA were occasionally detected in recipient oysters (<10^3^ copies mg^−1^ wet tissue; Fig. 1B). After infection, the level of OsHV-1 DNA increased above 10^4^ copies mg^−1^ in oysters irrespective of temperature, but rates of increase were highest at 21°C. At this temperature, the level of OsHV-1 amount exceeded 10^7^ copies mg^−1^ in one individual out of three at 24 hpc and in all oysters sampled at 48 hpc. This high value was reached 48 hpc at 29°C in only one individual out of three (Fig. 1B).

### Biochemical indicators of oyster physiology

We first characterized the initial physiological condition (0 hpc) of oysters at 21 and 29°C by means of biochemical analyses. Proximate composition (protein, lipid and carbohydrate) and CS activity, a proxy of tricarboxylic acid cycle activity and physiological condition (Dahlhoff, 2004) of oysters acclimated at 21 and 29°C, were remarkably similar (Dryad, Table S2; https://doi.org/10.5061/dryad.m37pvmcz0). In contrast, unsaturation index of polar lipids, an indicator of thermal adaptation of biological membranes, was higher at 21°C than at 29°C, mostly reflecting variations in 20:5n-3. Finally, the ratio of the fatty acids 20:4n-6 to 20:5n-3 increased with temperature.

### Sequencing and mapping

An average number of 15,699,960±4,059,920 millions of reads per library were obtained. All samples passed quality control measures for raw and trimmed sequence reads. After rRNA removal and trimming, about 76% of the reads mapped against the *C. gigas* reference genome. Numbers of raw reads, reads passing the quality filters, and uniquely mapping reads, are provided for each library in Table S1 (Dryad; https://doi.org/10.5061/dryad.m37pvmcz0).

### Transcriptomic responses among permissive and non-permissive interaction

**Fig. 2.**
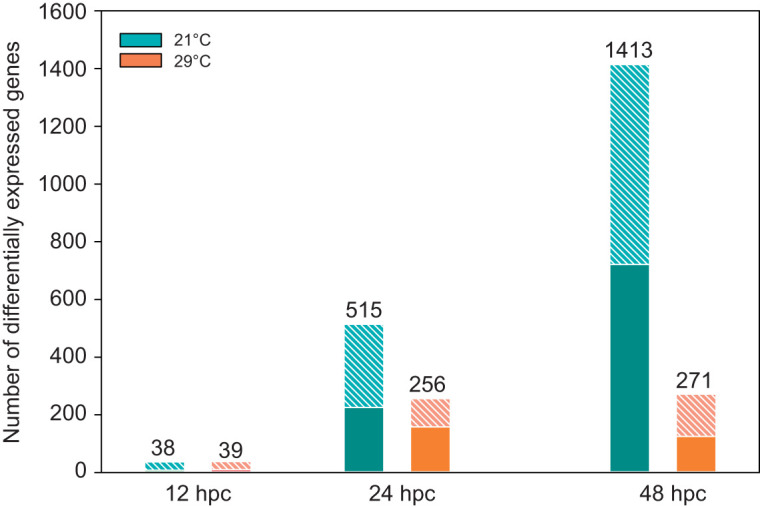
**Number of genes that are differentially expressed during OsHV-1 infection at 21 and 29°C compared with 0 hpc (reference).** The total number of differentially expressed genes is indicated above each bar. Up-regulated genes are represented as hatched bars and down-regulated genes as filled colored bars.

We then investigated the temporal transcriptomic response of oysters at 21°C and 29°C using 0 hpc as a reference point for each temperature. We found that the number of differentially expressed genes (DEGs) increased from 38 to 1413 between 12 and 48 hpc in oysters at 21°C while it remained low in oysters at 29°C (39 and 271 at 12 and 48 hpc, respectively, Fig. 2).

At 21°C and 12 hpc, the functional enrichment analysis revealed that up-regulated genes were associated with nine biological processes (BPs), all related to innate immune response (Dryad, Table S3; https://doi.org/10.5061/dryad.m37pvmcz0). At 24 hpc, 46% of the enriched BPs were related to immune response, e.g. cytoplasmic pattern recognition receptor signaling pathway in response to virus, cellular response to virus, positive regulation of interferon-alpha production (Dryad, Table S4; https://doi.org/10.5061/dryad.m37pvmcz0). Concomitantly, BPs related to negative regulation of necrosis, cell death, negative regulation of protein maturation and inhibition of the G2/M transition of mitotic cell cycle were enriched in up-regulated genes (Dryad, Table S4; https://doi.org/10.5061/dryad.m37pvmcz0). Among up-regulated genes at 48 hpc, a significant enrichment of gene related to innate immune response, negative regulation of cell death and negative regulation of protein maturation processes was observed. When analysing down-regulated genes, BPs related to growth, metabolic processes and regulation of cardiac muscle contraction were detected (Dryad, Table S5; https://doi.org/10.5061/dryad.m37pvmcz0).

At 29°C and 12 hpc, a significant number of up-regulated genes were associated with 19 BPs related to innate immune response (Dryad, Table S3; https://doi.org/10.5061/dryad.m37pvmcz0). At 24 hpc, down-regulated genes were enriched in BPs related to growth processes, cell development and transmembrane transport (Dryad, Table S4; https://doi.org/10.5061/dryad.m37pvmcz0). In addition, genes encoding apoptotic processes such as ‘lymphocyte apoptotic process’, ‘regulation of extrinsic apoptotic signaling pathway’ and ‘cell death’ were less abundant. The enrichment in genes involved in metabolic processes was confirmed at 48 hpc. BPs like ‘system process’, ‘endothelial cell development’, ‘regulation of anatomical structure morphogenesis’ and ‘divalent metal ion transport’ were over-represented among down-regulated genes, whereas those BPs related to innate immunity, ‘defense response to virus’ and ‘cellular homeostasis’ were over-represented among up-regulated genes (Dryad, Table S5; https://doi.org/10.5061/dryad.m37pvmcz0).

### Transcriptomic responses between permissive and non-permissive interactions

**Fig. 3.**
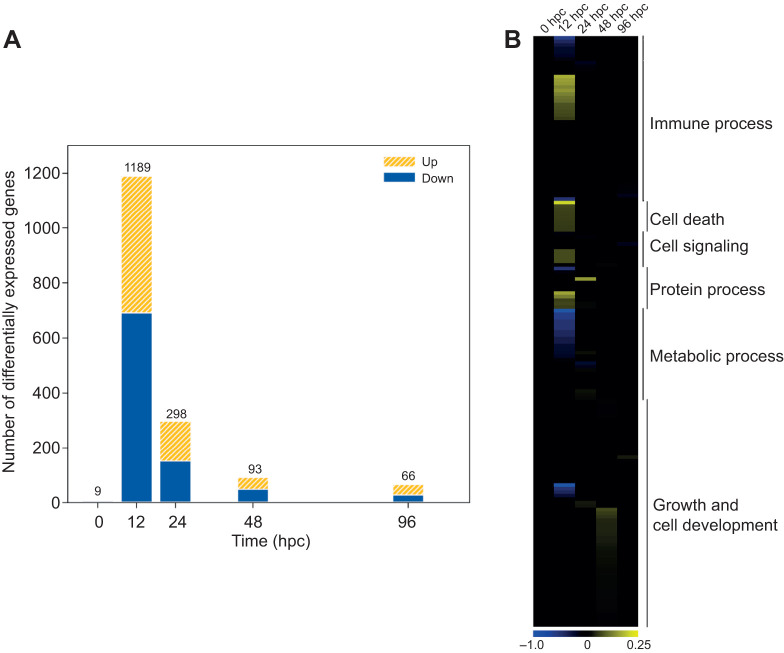
**Transcriptomic response of oysters infected at 29°C compared with their counterparts at 21°C.** (A) Number of genes that are differentially expressed at 29°C compared with 21°C (reference) over the time course of infection. The total number of differentially expressed genes is indicated above each bar. Up-regulated genes are represented in yellow and down-regulated genes in blue. (B) Heatmap of the 169 significantly enriched GO categories (*P*<0.05). The enrichment intensity was expressed as the ratio between the number of genes that were significantly up-regulated (yellow heat) or down-regulated (blue heat) in the category compared with the total number of genes in the category. If there is no significant difference in GO category enrichment between the tested conditions, the intensity was equal to zero (black heat). Details of the results (GO terms, enrichment values) are presented in Dryad (Table S13; https://doi.org/10.5061/dryad.m37pvmcz0).

We then compared the transcriptome of oysters infected at 29 and 21°C (reference) at each time point to identify key transcriptional events required for the repression of infection at 29°C (Fig. 3; Dryad, Table S6; https://doi.org/10.5061/dryad.m37pvmcz0). We found that oyster transcriptional profiles at 29 and 21°C showed only minor differences before infection as only nine DEGs were expressed. However, the number of DEGs increased to 1189 at 12 hpc and decreased to 298 at 24 hpc, 93 at 48 hpc and 66 at 96 hpc. Therefore, the effect of temperature on the transcriptional response of infected oysters appeared to be particularly strong at 12 hpc. For this reason, we focused the functional enrichment analysis on this point.

**Fig. 4.**
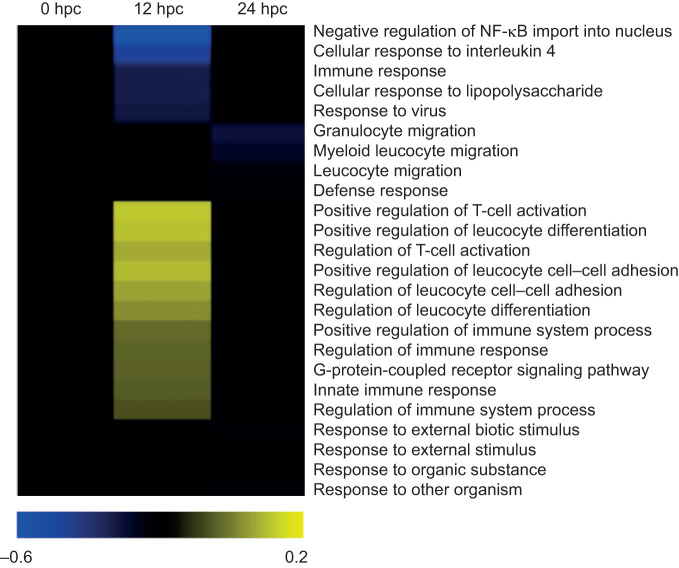
**Heatmap focusing on the 24 GO categories related to immune response that are significantly enriched (*P*<0.05) at 29°C compared with their counterparts at 21°C at 0, 12 and 24 hpc.** The enrichment intensity was expressed as the ratio between the number of genes that were significantly up-regulated (yellow heat) or down-regulated (blue heat) in the category compared with the total number of genes in the category. If there is no significant difference in GO category enrichment between the tested conditions, the intensity was equal to zero (black heat). Details of the results (GO terms, enrichment values) are presented in Dryad: Table S13 (https://doi.org/10.5061/dryad.m37pvmcz0).

The functional enrichment analysis revealed an enrichment of functional categories related to immunity at both temperatures (Figs 3B and 4). At 29°C, 11 functional categories related to immune response (e.g. positive regulation of T-cell activation, positive regulation of leucocyte differentiation, positive regulation of immune system process, regulation of immune response, innate immune response) were enriched at 12 hpc compared with only five enriched in the reference condition (21°C, negative regulation of NF-κB import into nucleus, cellular response to interleukin 4, immune response, cellular response to lipopolysaccharide, and response to virus; Fig. 4). Among the enriched functional categories related to immunity at 29°C (12 hpc), we identified some up-regulated genes that could be implicated in the OsHV-1 immune response like some pattern recognition receptors, intracellular signal transduction-TLR adaptor molecules, macrophage scavenger receptor and α2-macroglobulin molecule-like CD109 (Dryad, Table S7; https://doi.org/10.5061/dryad.m37pvmcz0). However, genes coding lectins, intracellular transduction TLR adaptor molecules, complement system component, intercellular signaling–TNF pathway or immune effectors were down-regulated at 29°C compared with the reference condition (21°C).

The functional enrichment analysis also showed that genes related to cell death, cell signaling and protein processes were up-regulated in oysters at 29°C compared with 21°C, whereas it was the opposite for those related to metabolism, growth and cell development (Fig. 3B; Dryad, Table S6; https://doi.org/10.5061/dryad.m37pvmcz0).

Up-regulated genes at 29°C related to cell death coded for 11 proteins involved in cell death *per se*, one autophagy protein and five apoptosis inhibitor proteins (Dryad, Table S8; https://doi.org/10.5061/dryad.m37pvmcz0). The 14 up-regulated genes related to cell signaling are mainly related to synaptic signaling and coded for neurotransmitter synaptic receptors such as acetylcholine, GABA, glycine, serotonin, thyrotropin releasing hormone and members of the glutamate pathway, neuropeptide receptor and G protein subunit alpha (Dryad, Table S9; https://doi.org/10.5061/dryad.m37pvmcz0). Finally, up-regulated genes at 29°C related to protein process coded for ubiquitin conjugating enzyme E2, E3 ubiquitin ligases proteins in charge of the substrate specificity (24 DEGs), and three deubiquitinase proteins were identified as regulators of this process (Dryad, Table S10; https://doi.org/10.5061/dryad.m37pvmcz0). Gene E3 is involved in regulation of apoptosis, protein translation arrest via EIF4E2 ubiquitination, degradation of misfolded proteins, regulation of immune response and major histocompatibility complex (MHC).

**Fig. 5.**
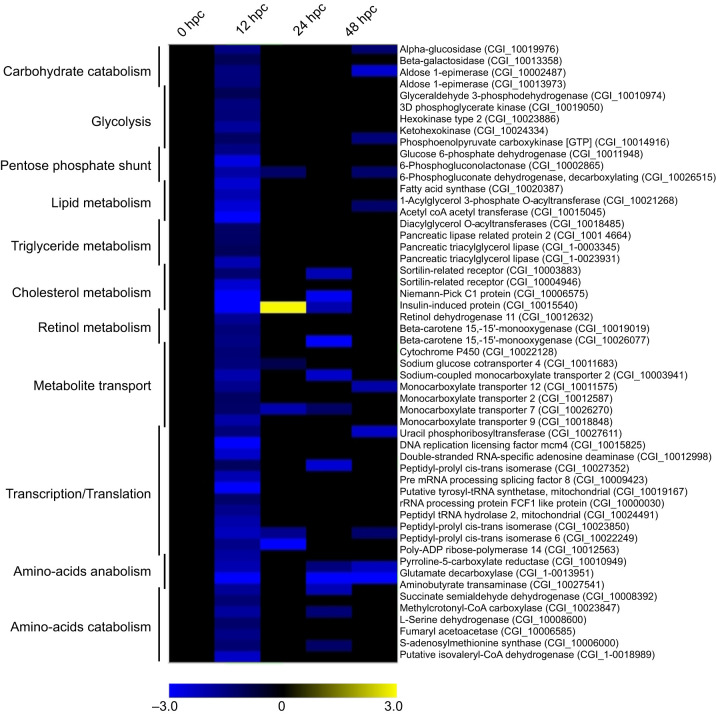
**Heatmap focusing on the 55 genes related to metabolic** **processes that were significantly down-regulated at 29°C, 12 h post-cohabitation.** The intensities of the colors indicate the magnitude of the differential expression (log2 fold-change). The fold-changes were calculated at each time point, comparing the expression of each gene at 29°C with its expression at 21°C. Details of the results (GO terms, enrichment values) are presented in Dryad, Table S13 (https://doi.org/10.5061/dryad.m37pvmcz0).

Down-regulated genes at 29°C related to metabolic processes coded for several proteins involved in catabolism of carbohydrate (4), amino acid (5) and triglyceride (4) and synthesis of fatty acids and phospholipid (3) and amino acids (3) (Fig. 5; Dryad, Table S11; https://doi.org/10.5061/dryad.m37pvmcz0). Genes coding for proteins involved in energy production pathways such as glycolysis (5), neoglucogenesis (1), pentose phosphate (4) and transport of monocarboxylate metabolites (5) were also down-regulated at 29°C. Finally, some down-regulated genes at 29°C were related to cholesterol metabolic process (5), retinol metabolism (5) and transcription and translation processes (11).

Thirty down-regulated genes at 29°C were also associated with processes like ‘regulation of growth’ and ‘cell adhesion’. These genes coded regulators of tissue growth (MEGF10, NOTCH1, CD63 antigen), and particularly for extracellular matrix components (collagen alpha chain, chondroitin sulfate troteoglycan, uromodulin; Dryad, Table S12; https://doi.org/10.5061/dryad.m37pvmcz0). Interestingly, the chondroitin sulfate proteoglycan was strongly down-expressed at 29°C (log2 fold-change, −11.09; *P*=1.37×10^−18^) like five members of the tetraspanin family, implying that these genes are associated with proteins involved in the entry of OsHV-1 into host cells. Other genes were involved in cell–cell adhesion (SVEP 1, contracted associated protein, Neurexin-2-alpha) and wounding repairs and inflammatory responses (Thrombospondin-1 and -2, Tenascin and Ninjurin-1). Most of these genes are associated with membrane glycoproteins that regulate numerous cellular functions such as cell extracellular matrix attachment, immunity and cell migration.

## DISCUSSION

**Fig. 6.**
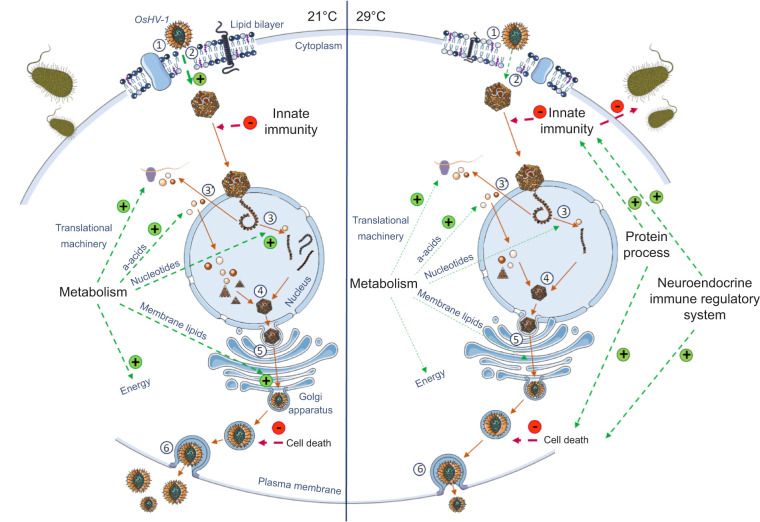
**Schematic representation of physiological responses occurring in oysters infected at 21 and 29°C** **at** **12 hpc.** This diagram is a data compilation of our results and the life cycle of OsHV-1 proposed by Jouaux et al. (2013). Font size increases for up-regulated processes and decreases for those that were down-regulated for the two tested temperatures. The green ‘+’ and the red ‘−’ arrows indicate the positive and negative effects of each process on oyster disease. Numbers refer to the different steps of OsHV-1 cycle: 1, viral attachment; 2, membrane fusion; 3, viral genome replication; 3′, transcription and translation of viral DNA; 4, capsid assembly; 5, envelopment of viral particle and releasing of mature virion. a-acids, amino acids.

In a previous paper, we found that survival of oysters challenged with OsHV-1 at 29°C was markedly higher than at 21°C, whereas virus infectivity and virulence were unaltered (Delisle et al., 2018). Here we investigate how temperature influenced the host response to the pathogen by comparing transcriptional profiles by means of RNA sequencing during the course of infection at 21 and 29°C. We found that temperature affected the expression of genes related to immune processes, cell death, synaptic signaling, protein processes, metabolism, growth and cell development (Fig. 3). These processes occur in all stages of infection (from 12 to 96 hpc) and are summarized in Fig. 6.

Prior to infection, oysters at 21 and 29°C showed only nine DEGs, suggesting that they were fully acclimated after 10 days. This is consistent with the fact that filtration rates of oysters (Delisle et al., 2018) and their proximate composition and aerobic metabolism (CS activity) were similar at both temperatures. Also, the unsaturation index of polar lipids, an indicator of thermal adaptation of biological membranes, varied consistently with temperature (Hochachka and Somero, 2002; Pernet et al., 2007).

The number of DEGs varied from 38 at 12 hpc to 1413 to at 48 hpc, depending on temperature and time. In previous studies investigating the response of oysters to OsHV-1 infection, the number of DEGs varied from 250 to 9400 (De Lorgeril et al., 2018; Jouaux et al., 2013; Rosani et al., 2015). The relatively low number of DEGs obtained here probably reflects that we compared transcriptomes of both susceptible and infected oysters at two temperatures that exhibited subtle differences in disease severity.

The number of DEGs in oysters infected at 21°C, compared with relative controls, increased markedly throughout the duration of the study, whereas it remained low and stable at 29°C. A similar trend has been reported when comparing transcriptomes of oyster families with contrasted resistance phenotypes with regard to the disease (De Lorgeril et al., 2018). For instance, the number of DEGs increases markedly during the time course of infection in the susceptible oyster family, whereas it remains low and stable in the resistant family (De Lorgeril et al., 2018). Therefore, from a quantitative point of view, the temporal transcriptomic response of oysters infected at 21 and 29°C resembles that of susceptible and resistant families, respectively.

Our results suggest that seawater temperature could modulate the entry of OsHV-1 into the host cell by modulating the composition of the cell matrix and the attachment of the virus in a way consistent with a reduced susceptibility of the host to the virus at 29°C. For instance, the gene coding for chondroitin sulfate proteoglycans was down-regulated at 29°C compared with 21°C at 12 hpc. This protein is a major component of the extracellular matrix of vertebrates, which, among other things, participates in the attachment of the herpes simplex virus 1 to the host cell (Mårdberg et al., 2002). In *C. gigas*, chondroitin sulfate proteoglycans are particularly abundant in gills (Zhang et al., 2012), which is the entry portal of pathogens like OsHV-1 (Martenot et al., 2016; Segarra et al., 2016). Previous studies suggested that heparan sulfate, another extracellular matrix component, could be implicated in OsHV-1 entry in oyster cells (Jouaux et al., 2013; Segarra et al., 2014a). We also found that five tetraspanins, a family of transmembrane proteins, were less abundant at 29°C. Notably, among these proteins, the *CD63* gene codes for an antigen involved in the entry of several types of viruses into host cells (Spoden et al., 2008; Van Spriel and Figdor, 2010).

Although viral loads reached values above 10^7^ copies mg^−1^ 24–48 h after the onset of infection at both 21 and 29°C, expressions of some viral genes were lowered and viral infection failed to induce mortality at 29°C (Delisle et al., 2018). We found that temperature modulates oyster immunity as reported by Meistertzheim et al. (2007). For instance, at 29°C, 11 functional categories related to immune response were enriched and genes coding for pattern recognition receptors were up-regulated. Temperature increases the expression of genes related to immunity such as TLRs, RIG-I and tumor necrosis factors in healthy oysters (Meistertzheim et al., 2007; Zhang et al., 2015; Lafont et al., 2020).

In accordance with the RNA sequencing, the ratio of the fatty acids 20:4n-6 to 20:5n-3 increased with temperature, suggesting that the immune status of oysters was more favorable at 29°C. Indeed, these two fatty acids are involved in eicosanoid production, which is associated with stimulation of immune function in invertebrates (Howard and Stanley, 1999). However, eicosanoids produced from 20:4n-6 are generally more active than those produced from 20:5n-3, and the replacement of 20:5n-3 by 20:4n-6 in bivalves decreases immune parameters of hemocytes (Delaporte, 2003; Delaporte et al., 2006, 2007). Despite these results, up-regulation of genes known to be involved in anti-viral defense against OsHV-1 were not highlighted at 29°C. This might be consistent with the hypothesis that efficient anti-viral response at 29°C has occurred earlier than 12 hpc as suggested by De Lorgeril et al. (2018) where a strong anti-viral response arose 6 h post-infection in resilient oysters. These results might also suggest that high survival of oysters infected at 29°C could be based on other processes such as apoptosis.

We found that temperature increased the expression of pro-apoptotic genes in oysters at 12 hpc. Apoptosis is one of the major mechanisms of anti-viral response inducing the abortion of viral multiplication and the elimination of viral progeny by premature lysis of infected cells (Pilder et al., 1984; Segarra et al., 2014b). Therefore, up-regulation of some pro-apoptotic genes at 29°C may have limited virus proliferation, as previously reported in shrimps exposed to high temperature treatment during a viral infection (Granja et al., 2003). At the same time, some genes coding for apoptosis inhibitors were also up-regulated at both 21 and 29°C. The role of apoptosis during OsHV-1 infection is still unclear (De Lorgeril et al., 2018; Rosani et al., 2015; Segarra et al., 2014a; Wang et al., 2018). However, the outcome of the disease seems to rely on a finely tuned balance between apoptotic and anti-apoptotic effects, sometimes even triggered by the virus (Green et al., 2015).

Temperature also influenced the ubiquitylation and proteasome system of oysters. For instance, the number of DEGs coding for the ubiquitylation and proteasome system increased at 29°C and 12 hpc. Similarly, previous studies showed that the number of DEGs related to the ubiquitylation process increased during the first 6–12 h post-infection and was higher in resistant than in susceptible oysters (De Lorgeril et al., 2018, 2020). Moreover, the amount of protein related to the ubiquitination process was altered in infected oysters (Corporeau et al., 2014). In vertebrates, ubiquitylation and proteolysis are involved in protein recycling and regulate the stability, activity and localization of target proteins (Bhoj and Chen, 2009). Several up-regulated genes identified in our study belong to protein E3, which is involved in major histocompatibility complex, apoptosis, cell cycle and protein translation arrest. These processes are crucial for mounting an adequate immune response and are likely to fight against bacterial pathogens (Cheng et al., 2016; Seo et al., 2013). It is therefore probable that over-stimulation of the proteasome system in oysters at 29°C could limit the bacteremia that normally follows OsHV-1 infection and leads to oyster death (De Lorgeril et al., 2018).

We found that genes related to several neurotransmitter synaptic receptors were up-regulated in oysters infected at 29°C and 12 hpc. These genes coded for receptors for acetylcholine, GABA, glutamate, serotonin, thyrotropin-releasing hormone and gonadotropin-releasing hormone. Oyster hemocytes generally have receptors for several of the previously listed neurotransmitters. Hemocytes regulate receptor abundance during the immune response to influence apoptosis and phagocytosis, indicating that, once the host recognized an invader, some neurotransmitters might be released to optimize immune responses (Li et al., 2016; Liu et al., 2017). In oysters, the release of GABA and acetylcholine seems to be activated by immune stimulation. Over a long time scale, they tend to down-regulate the immune response, avoiding an excess of immune reactions in order to maintain immunity homeostasis (Li et al., 2016; Liu et al., 2017; Wang et al., 2018). At 29°C, over-expression of neuroendocrine receptors could optimize immune activities, helping to eliminate OsHV-1 and to restore homeostasis after viral elimination.

In response to viral infection at 29°C, genes related to catabolism, metabolites transport, amino acids, nucleotides and fatty acids synthesis, transcription and translation were down-regulated more at 29°C than at 21°C in our study. This probably reflects that OsHV-1, like other herpesviruses, alters host cell metabolic pathways to provide an optimal environment for its replication and spread (De-la-Re-Vega et al., 2017; Thaker et al., 2019). More particularly, viruses increase (i) nucleotide synthesis to supply rapid viral genome replication, (ii) amino acid production used for virion assembly, (iii) lipid availability to provide additional membrane material for envelopment of viral particles and (iv) ATP flux for supporting the energy cost of genome replication and packaging (Sanchez and Lagunoff, 2015; Yu et al., 2011). More specifically, OsHV-1 increases glycolysis, TCA cycle, and fatty acid synthesis and triglyceride catabolism, indicative of host metabolic changes related to viral infection (Corporeau et al., 2014; Young et al., 2017). Other studies report depletions of host carbohydrate and triglyceride reserves during OsHV-1 infection (Pernet et al., 2019). Here we showed that potential hijacking of the host cell machinery by OsHV-1 was altered at 29°C.

### Conclusion

To conclude, this study provides new insights into the mechanisms underlying the high survival of oysters infected with OsHV-1 at high temperature (29°C), as demonstrated previously (Delisle et al., 2018). As summarized in Fig. 6, temperature induces alterations in the membrane composition, limiting the entry of OsHV-1. At 29°C, immune response and up-regulation of genes encoding for apoptosis result in the limitation of early viral replication. At the same time, down-regulation of genes encoding for catabolism, metabolite transport, macromolecule synthesis and cell growth reduces the availability of cellular components essential to viral particle synthesis. Finally, up-regulation of genes encoding proteasome processes and synaptic signaling associated with the limitation of viral replication seems to have prevented bacteremia and limited oyster mortality. Taken together, these transcriptomic responses can explain the increased robustness of infected oysters at 29°C.
